# The Role of the Biosafety Cabinet in Preventing Infection in the Clinical Laboratory

**DOI:** 10.7759/cureus.51309

**Published:** 2023-12-29

**Authors:** Gaurav A Jagtap, Ankit Badge, Mangesh G Kohale, Rashmi S Wankhade

**Affiliations:** 1 Pathology, School of Allied Health Sciences, Datta Meghe Institute of Higher Education and Research, Nagpur, IND; 2 Microbiology, Datta Meghe Medical College, Datta Meghe Institute of Higher Education and Research, Nagpur, IND; 3 Pathology, Datta Meghe Medical College, Datta Meghe Institute of Higher Education and Research, Nagpur, IND

**Keywords:** aerosols, laboratory safety, microorganism, biosafety cabinet, pathogen

## Abstract

Clinical laboratories are essential in healthcare to better diagnose, treat, and track medical diseases. However, handling infectious organisms and possibly infectious materials in these laboratories puts the safety of laboratory workers and the general public at risk. By controlling the distribution of infectious substances and stopping the spread of diseases, biosafety cabinets (BSCs) have become crucial tools in guaranteeing laboratory safety. The prevention of infections is most important in medical and laboratory settings. In clinical laboratories, biological and infectious agents are handled, posing threats to healthcare workers and the general public. To avoid infections, proper training of the BSC is essential. Laboratory employees are instructed in aseptic procedures, proper hand posture, and efficient personal protection when working in the cabinet. These instructions decrease the chance of contaminating the surrounding area. Additionally, user ergonomics are taken into account while designing BSC, reducing operator fatigue, and guaranteeing that staff can execute tasks precisely for extended periods. This review highlights the importance of biosafety cabinets in maintaining a secure laboratory environment and explains their crucial function in infection control.

## Introduction and background

Biosafety is essential for the management of clinical laboratories to stop the introduction and spread of infection. Biosafety measures defend against the potential destruction caused by new diseases by implementing strict regulations, safety precautions, and research practices [1]. Fundamentally, biosafety is the management of biological materials to protect people, the community, and the environment. It includes a variety of actions, rules, and guidelines designed to stop the unintended release of hazardous substances or accidental exposure to them. Bacteria, viruses, fungi, poisons, and other potentially dangerous microbes can be among these agents [2]. Researchers, healthcare professionals, and others reduce the danger of infections caused by these substances by following stringent biosafety standards. Laboratories are centers of scientific discovery and study but also carry the risk that infectious pathogens could accidentally be released [3]. Based on the possible dangers they manage, laboratories are classified using the biosafety level (BSL) system. More harmful agents are handled with higher BSL. Laboratories have strong biosafety cultures that guarantee the correct use of personal protective equipment (PPE), regulated access, and efficient waste management. Such precautions reduce the risk of spreading infections, protecting researchers and the general public [4].

Maintaining a controlled and safe laboratory environment to prevent infections is paramount, especially in facilities that handle hazardous biological materials. Biosafety cabinets (BSCs) play a critical role in ensuring the safety of laboratory personnel and the surrounding environment and the integrity of experimental work involving biological agents. Proper maintenance, calibration, and use of biosafety cabinets have a significant impact on the achievement of safety objectives [5]. Advanced BSCs have digital touchscreen control, remote monitoring capabilities, and data logging, which allow laboratory managers to monitor cabinet performance, track usage, and address issues that contribute to overall safety. Advanced lighting systems using light-emitting diode (LED) technology improve visibility within the cabinet [6]. Proper illumination improves accuracy and minimizes mistakes while working with potentially hazardous materials. BSCs include built-in interactive training modules that guide users in appropriate techniques and safety protocols. This helps prevent errors and ensures consistent adherence to best practices [7]. Ensuring that all laboratory staff are adequately trained and competent in using BSCs is a challenge. Improper use and technique can compromise the effectiveness of the BSC and lead to potential contamination. Insufficient training can lead to human errors, such as incorrect hand placement, accidental movements that alter airflow, and failure to maintain aseptic conditions [8]. This study aims to elucidate the critical role of BSCs in preventing infection transmission in clinical laboratory settings by exploring their design features and operational protocols.

## Review

Method

To conduct a review literature search, we used the following databases: PubMed, Scopus, and Google Scholar. We searched for articles published between 2011 and 2023. The following search terms were used: (pathogen) AND (Biosafety cabinet) OR (infectious agent) AND (infective agent) AND (aerosol) AND (Advancement) AND (cleaning) OR (decontamination) AND (microorganism). We applied the following inclusion criteria for the final review: (1) original research article, (2) English language, (3) peer-reviewed, (4) role of biosafety cabinet in the clinical laboratory to prevent infection, (5) full-text available, and (6) published in the specified time frame.

Articles Screened

**Figure 1:**
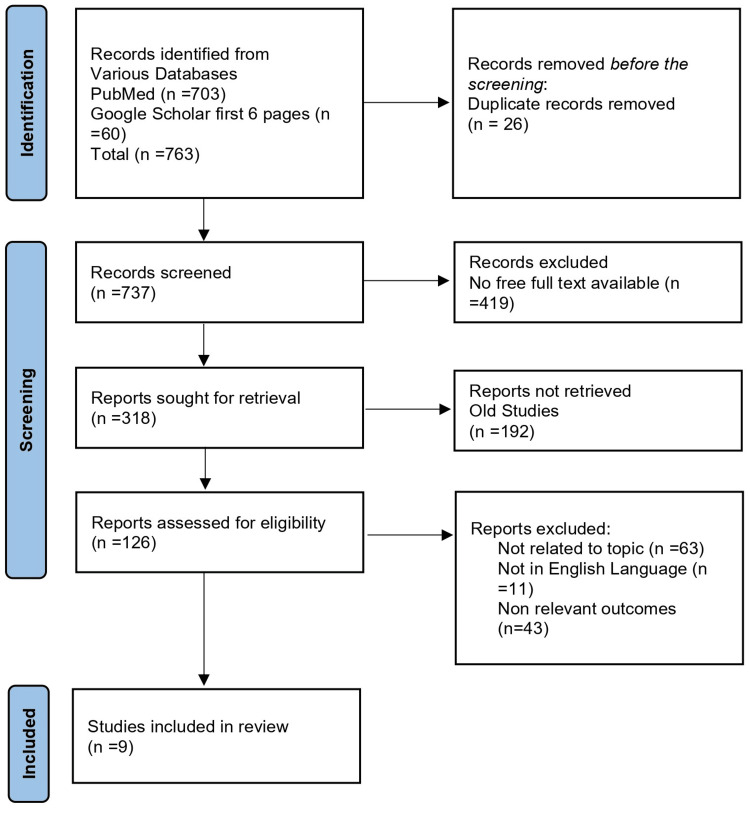
PRISMA flow chart n: Number of studies; PRISMA: Preferred Reporting Items for Systematic Reviews and Meta-Analyses

After conducting the initial search, we identified a total of 763 articles in the searched database. We excluded 26 duplicates and conducted an initial screening of the remaining articles; we excluded 419 articles after the full-text screening of the remaining 737 articles. We excluded 192 articles that were old studies before 2018 and 117 articles that were not related to the topic, not in the English language, and did not include relevant outcomes. We included nine articles for the final review from 2018 to 2022 (Figure 1).

The final review included a total of nine articles from the years 2018 to 2022 (Table 1). 

**Table 1:** Studies included in the review PPE: Personal protective equipment; COVID-19: Coronavirus disease of 2019; SARS-CoV-2: Severe acute respiratory syndrome coronavirus 2

Sr. no.	Author	Year	Conclusion	Finding
1	Zhou et al. [9]	2022	Improved biosafety measures and optimized workflow in clinical microbiology laboratories and prevention and control effect on preventing infection of medical staff.	Biosafety measures are effective protection against infections among medical staff. These measures help reduce the risk of infection, promote good working habits, and provide guidelines for biosafety practices in microbial laboratories.
2	Bhat et al. [10]	2020	Laboratory biosafety measures minimize the risk of COVID-19 transmission to healthcare workers and proper specimen handling is important at all levels.	Handling of patients diagnosed with COVID-19 and airborne precautions for procedures that generate aerosols.
3	Pentella [11]	2020	Clinical laboratories are unprepared for biosafety challenges and clinical laboratory leaders should build a biosafety program.	Biosafety program that clinical laboratory leaders should build to meet the needs of clinical laboratories; biosafety challenges of automated laboratory systems.
4	Gassiep et al. [12]	2021	No infections or seroconversion occurred during routine handling. Risk assessment and guidelines for specific regions should be considered.	The risk of laboratory-acquired melioidosis is low. However, individual laboratories will need to undertake a risk assessment.
5	Kaufer et al. [13]	2020	The interim classification of SARS-CoV-2 should be accepted and finalized to inform the appropriate biosafety levels, physical containment facilities, and PPE required for the handling of SARS-CoV-2 and to minimize the risk of laboratory-acquired COVID-19 infections during this public health emergency.	Biosafety precautions, control measures, and appropriate physical containment facilities are required to minimize the risk of laboratory-acquired infections with SARS-CoV-2.
6	Cornish et al. [14]	2021	Biosafety gaps in clinical laboratories need to be addressed. Collaborative solutions are needed to improve biosafety.	The need for collaborative, comprehensive solutions to improve clinical laboratory biosafety and to better combat future emerging infectious disease outbreaks.
7	Gardezi et al. [15]	2020	Biosafety practices are crucial in laboratory analysis of clinical samples from COVID-19 patients. Poor resource settings require cost-effective and improvised solutions for the safe handling of samples.	The correct use of PPE and its suitable alternatives are available for selection and use. Disinfection of the lab areas and safe disposal of the clinical samples from such patients is also of paramount importance.
8	Peng et al. [2]	2018	Laboratory-acquired infections pose significant health risks. Proper biosafety and biosecurity measures are necessary to prevent these infections.	Laboratory-acquired infections and related risks. Preventive strategies to tackle laboratory-acquired infections.
9	Wang et al. [16]	2020	Biosafety prevention and control measures are necessary.	Provides practical suggestions for laboratory staff to avoid laboratory-acquired infections in dealing with public health emergencies.

Out of the nine articles included in Table 1, four studies [9,10,13,16] focused on biosafety prevention and control measures. Three studies [2,11,12] examined biosafety programs and risk assessment. The improved clinical biosafety in the laboratory and the ability to better combat future emerging infectious disease outbreaks are explained in one study [14]. Another study [15] showed the importance of biosafety practices.

BSCs are crucial in clinical laboratories to maintain the health of laboratory personnel. BSCs help to protect laboratory personnel from bacteria, viruses, and other diseases [17]. A BSC maintains a clean working environment by filtering the air with high-efficiency particulate air (HEPA) filters. A BSC also provides a physical barrier between employees and samples, reducing direct contact with harmful compounds [18]. By preserving aseptic conditions, a BSC safeguards laboratory workers and prevents the spread of diseases, promoting a safe and secure work environment for all individuals participating in clinical testing [19]. BSCs can protect laboratory workers from infections in clinical settings. These cabinets provide a controlled, enclosed environment that prevents infectious organisms from spreading throughout the lab [20]. They successfully eliminate infectious particles, bacteria, and viruses from the air by employing HEPA filters and decrease the possibility of personnel contracting diseases by preventing unintentional inhalation or exposure to hazardous materials [21]. A BSC ensures aseptic procedures during procedures, reducing the possibility of cross-contamination. Additionally, its containment abilities stop the transfer of infectious agents outside the work area, protecting both laboratory personnel and the surrounding area. BSCs are essential to ensure a secure work environment and protect lab workers from infection risks [22]. BSCs are classified as class I, class II, and class III [23].

Class I biosafety cabinet

The environment and people are protected by a class I BSC; however, samples or materials are handled inside the cabinet. This type of BSC has vertical laminar airflow and an open front. A class I BSC is typically used when working with low- to moderate-risk agents that do not require sterile conditions. These BSCs are appropriate for situations where containment is the primary need, but sterility is not necessary because they provide only minimal protection against aerosols and splashes. Class I BSCs enable the secure handling of liquid samples for early processing, such as centrifugation and decanting. These cabinets are commonly used in research laboratories and pharmaceutical industries for tasks such as handling and weighing powders, mixing chemicals, and transferring cultures, allowing researchers and scientists to work safely with hazardous substances. Class I BSCs are essential to ensure personnel safety and prevent the spread of contaminants. Distilled water, benzalkonium chloride (BKC) with corrosion inhibitor, ethanol, peracetic acid, hydrogen peroxide, and ultraviolet (UV) radiation are commonly used to maintain the sterility of class l BSCs before and after sample processing [24].

Class II biosafety cabinet

The cabinets used most often in clinical laboratory settings are class II BSCs. Class II BSCs are designed to provide protection for both personnel and products. These BSCs have a front opening and vertical laminar airflow, which forms a barrier to protect samples from contamination. These BSCs also have HEPA filters, which provide a sterile environment for delicate items by removing airborne particles and bacteria. A1, A2, B1, and B2 are four subtypes of class II cabinets and each has unique airflow patterns and exhaust devices. Airflow patterns in class II BSCs ensure that any contaminants generated during the process are captured and filtered out, preventing their release into the surrounding environment. These BSCs should be regularly cleaned with disinfectants to prevent contamination. Annual calibration by professionals, checking airflow, HEPA filters, and cabinet integrity, should be conducted [25]. Regular inspection of seals, training of users on safety protocols, and the maintenance of detailed records for compliance should be undertaken. Applications that require both containment and sterility, such as cell culture, molecular testing, virus handling, and other processes, are best served by class II BSCs [26].

Class III biosafety cabinet

Glove boxes, commonly referred to as class III BSCs, are the highest level of containment and are made to handle extremely contagious agents. Class III cabinets include integrated glove ports and are entirely enclosed, allowing for the manipulation of items inside. All materials are passed through airtight ports while personnel work with the samples while wearing connected gloves. The airflow is tightly managed to stop any harmful items from escaping. Maximum containment laboratories employ class III BSCs, which are appropriate for handling extremely infectious viruses and pathogens [27]. The best type of BSC to use in clinical laboratory settings depends on the particular tasks at hand, the level of containment required, and the infection involved in the handling of the chemicals. Because they provide both containment and sterility, class III BSCs are the most adaptable and frequently used, making them suitable for a variety of applications. Pathogenic samples such as sputum samples for tuberculosis (TB), scabs or bodily fluid samples for smallpox, blood or tissue samples for hantaviruses, and respiratory secretion samples for influenza are handled in class III BSCs. HEPA filters capture tiny particles, including harmful microorganisms, making the filtered air cleaner than its surroundings. To prevent any risk, laboratories that use HEPA filters have special negative pressure rooms. These rooms are designed to keep filtered particles inside. HEPA filters allow air to flow in but not out, reducing the chance that any released particles reach the outside and cause harm [28]. In cell growth, nucleic acid manipulation, and general microbiological work environments, this type of BSC is perfect. Higher containment is necessary for activity with infectious agents [29].

Physical design and airflow

The purpose of a BSC is to create a physical separation between laboratory workers and the products they are handling. BSCs include a front aperture with a transparent window to give a view and access to the work area. Typically, the airflow inside the cabinet is unidirectional, moving from the top to the front. Through HEPA filters, this design ensures that pollutants produced during processes are removed from personnel. This ensures that lab workers are protected from potential contamination and exposure to hazardous substances. Additionally, the airflow design helps maintain a clean and sterile environment within the cabinet, reducing the risk of cross-contamination between different samples or experiments. HEPA filters further enhance safety measures by effectively capturing and removing airborne particles, promoting a healthier and safer working environment for laboratory personnel [30].

HEPA Filters

BSCs must have HEPA filters. With a 99.99% efficiency rate, these filters can collect and eliminate particles as small as 0.3 microns. HEPA filters efficiently capture bacteria, viruses, and other infectious particles, preventing their escape into the laboratory environment and protecting laboratory staff from exposure. In addition to protecting lab staff, HEPA filters also play a crucial role in maintaining the integrity of experiments and research. By preventing contamination from external sources, the filters ensure the accuracy and reliability of data generated within the lab. Furthermore, the use of HEPA filters for BSCs is essential for meeting regulatory requirements and maintaining a safe working environment, especially in laboratories that work with hazardous materials or biological agents. These filters help to create a controlled environment by filtering out airborne particles and microorganisms, reducing the risk of cross-contamination. This is particularly important in laboratories that handle sensitive samples or perform research on infectious diseases. Additionally, the use of HEPA filters can also extend the useful life of delicate equipment by preventing dust and debris from clogging or damaging sensitive components [31].

Inward and Downward Airflow

In addition to maintaining a sterile environment inside the cabinet and preventing unfiltered air from entering the workspace, the downward airflow within the BSC aids in the creation of a personal safety zone for the laboratory worker by preventing contaminants from escaping the work area. This personal safety zone is crucial in ensuring the protection of the workers and maintaining the integrity of the experiments being conducted. The downward airflow also helps prevent contamination between the samples and the environment, further enhancing the reliability of the results obtained [32].

Safe Work Practices

Aseptic methods, such as avoiding airflow disturbances and eliminating clutter to promote smooth airflow are required for the correct use of BSCs. To further limit the risk of contamination, personnel should wear the appropriate personal protective equipment [33]. Continual maintenance and certification are needed to guarantee the best performance and safety. Regular filter replacement, airflow monitoring, and certification by trained experts help spot and resolve problems that can risk the containment capabilities of the BSC [34]. When handling hazardous materials within the BSC, proper disinfection procedures are crucial. To avoid cross-contamination, the work surface and the interior of the cabinet should be cleaned and disinfected both before and after each use. In BSCs, flames can obstruct ventilation and create a fire hazard; open flames or candles should never be used within a BSC [35].

Operational Protocols

Operational protocols for the safe use of a BSC are of paramount importance. Adhering meticulously to established safety procedures is imperative to ensure both personal safety and the integrity of research results. To begin with, proper placement of hands inside the cabinet is essential to maintain the prescribed airflow and prevent potential cross-contamination. Before beginning work, thorough sterilization procedures must be observed, including comprehensive cleaning and disinfection to eliminate any residues from previous sessions [14]. Maintaining a clutter-free workspace is crucial, as it minimizes air disturbances and promotes a consistent airflow pattern within the cabinet. Wearing appropriate personal protective equipment, such as gloves, lab coats, and safety goggles, is not negotiable for laboratory personnel working with BSCs to prevent exposure to hazardous substances [36]. Limiting the duration of work within the BSC is advisable, reducing the chances of mishaps and distractions that could lead to contamination. After work, meticulous decontamination of all internal surfaces is essential to preserve a sterile environment for subsequent sessions of infectious agents. Regular maintenance and certification, performed by certified personnel, are essential to ensure ongoing compliance with safety regulations and the optimal functioning of the BSC [37].

Aerosols and particle containment

HEPA filters are used by BSCs to purge the working environment of airborne dust and aerosols. These filters have a 99.97% efficiency in trapping germs and particles as small as 0.3 microns. A BSC considerably lowers the cross-contamination between samples and specimens during handling and manipulation by confining these potential pollutants [38].

Suitable Work Methods

To protect the integrity of the sterile workspace, the BSC is constructed with particular standards for hand positioning and instrument placement. To maintain the isolation of the sample and minimize the risk of cross-contamination, laboratory staff are trained to perform their duties within the BSC using these appropriate work practices. These practices include wearing appropriate personal protective equipment such as gloves, lab coats, and face masks. Additionally, staff are instructed to avoid unnecessary movements or reaching over the open workspace to prevent potential contamination. Regular cleaning and disinfection of the BSC are also essential to ensure a sterile environment and reduce the risk of contamination. By adhering to these guidelines, the laboratory can ensure accurate and reliable results while protecting the health and safety of both the staff and the samples being handled [30,39].

Physical Barriers

The clear BSC front window acts as a physical barrier preventing laboratory staff from coming into contact with samples or specimens directly. Unintentional spills or contact with hazardous materials that could cause cross-contamination are decreased by this barrier. A 15-minute exposure to ultraviolet (UV) germicidal lamps is a feature of a BSC that allows laboratory personnel to thoroughly remove bacteria that accumulate on surfaces by decontaminating the workstation before and after use. A BSC offers an additional line of defense against cross-contamination by exploiting this feature. This lamp emits UV light, which is known to kill or inactivate a wide range of microorganisms. By effectively sterilizing the workspace, the UV germicidal lamp adds an additional layer of protection in preventing the spread of harmful bacteria or viruses. With the use of a BSC and its UV germicidal lamp, laboratories can ensure a safer environment for both their staff and the samples being handled [40].

Cleaning and Decontamination

To keep a sterile workspace, the BSC must be cleaned and decontaminated regularly. Cross-contamination between clinical samples is reduced when proper disinfection procedures are performed to remove any potential leftover pollutants. Reducing the time employees spend working in the BSC helps avoid unintentional contamination caused by prolonged exposure to potential pollutants. Shorter working periods increase the reliability of laboratory results and reduce the possibility of cross-contamination. This not only improves the reliability of laboratory results but also significantly decreases the probability of cross-contamination, ensuring accurate and reliable scientific results [41]. A BSC is used when working with hazardous and infectious agents or volatile chemicals. By isolating these materials within the cabinet, the risk of spreading contamination to other clinical samples or specimens in the laboratory environment is minimized [42]. The calibration and proper use of BSCs to maintain a controlled and safe laboratory environment and prevent infections are of great significance. These aspects are critical to ensure the effectiveness of BSC in containing and controlling infectious agents, protecting laboratory personnel, and preventing the spread of infections. Without proper calibration and maintenance, the laboratory environment can become a breeding ground for infectious diseases, compromising the safety of researchers and the general public. Regular checks and adjustments are necessary to ensure that the BSC functions correctly and provides the necessary level of containment. By adhering to strict protocols and implementing preventive measures, laboratories can minimize the risk of infection and maintain a safe working environment for all the personnel involved [43].

Containment of infectious agents

The correct operation of all parts, including HEPA filters and airflow systems, is guaranteed by the routine maintenance of the BSC. Infectious agents can be efficiently contained within the workspace by well-maintained cabinets, avoiding their release into the laboratory environment and reducing the risk of contamination. [12]. When the BSC is calibrated correctly, the airflow patterns are ideal, resulting in a stable and controlled sterile environment. The laminar airflow is maintained in well-calibrated cabinets, successfully protecting samples and specimens from airborne pollutants and lowering the risk of cross-contamination. Self-closing sashes and UV germicidal lamps are examples of safety measures that benefit from weekly maintenance and annual calibration. These characteristics increase overall lab safety by shielding laboratory personnel from unintentional exposure to harmful materials [44]. The risk of contamination of samples and specimens is reduced by using BSCs correctly, according to the positioning instructions, and minimizing unnecessary movement. By following these procedures, lab staff can work securely inside the cabinet and avoid accidentally contaminating delicate items.

The interior of the BSC is kept sterile through routine maintenance, including extensive cleaning and disinfection [45]. Routine maintenance reduces the chance of unexpected breakdowns and equipment failures. Preventive actions, such as filter replacement and component inspections, keep BSCs in top condition, reducing downtime and guaranteeing ongoing protection for laboratory staff [46]. BSCs must be properly maintained and calibrated to adhere to regulatory norms and guidelines. By adhering to these regulations, the laboratory operates safely and according to accepted industry standards, preventing potential fines or legal problems [47]. The longevity of a BSC is increased by routine maintenance and annual calibration. The effectiveness of well-maintained cabinets lasts longer and continues to safeguard the lab against contamination. The laboratory can save money by avoiding unexpected repairs or replacements by keeping the BSC properly calibrated and maintained. Using resources effectively enables the lab to commit funds to other crucial research and safety projects [48]. The BSC must be correctly calibrated and maintained to offer a consistent and reliable working environment for laboratories. The accuracy of the results, which is essential for patient diagnoses and treatment choices, may be relied on by researchers and staff. The risk of infection transmission to laboratory personnel is considerably reduced by the combined effects of BSC maintenance and calibration; this makes the workplace safer [49]. Best practices for BSC maintenance and operation are as follows (Table 2).

**Table 2:** Stepwise practices for BSC maintenance and operation BSC: Biosafety cabinet; HEPA: High-efficiency particulate air; PPE: Personal protective equipment

Step	Action
Training	Ensure personnel have proper training in BSC operation and safety procedures [7].
BSC inspection	Check the BSC for physical damage, leaks, and proper airflow. Verify that the HEPA filters are intact and functioning [18].
Calibration	Schedule annual calibration of the BSC with a certified technician. Confirm airflow velocity and patterns meet safety standards [44].
BSC operation	Wear appropriate personal PPE, including gloves and a lab coat. Disinfect the BSC interior surfaces before and after each use [35].
Risk reduction	Minimize movement within the BSC to avoid disrupting airflow. Avoid blocking front and rear grilles to maintain proper airflow [31].
Post-operation	Allow the BSC to run for a few minutes after work to clear the air. Disinfect the BSC interior surfaces again. Remove and properly dispose of contaminated materials [43].
End	Record BSC maintenance, calibration, and operation details for documentation [48].

Challenges and limitations 

Budgets for laboratories may be a challenge as BSCs can be expensive to buy and maintain because they need regular filter replacement and calibration. Inappropriate use, including leaving the cabinet open or not adhering to the correct rules, might weaken containment and cause infections [50]. Since clinical laboratories frequently have limited space, it may be difficult to accommodate BSCs, which affects where they are placed and how effectively airflow occurs. Improperly cleaned and decontaminated BSCs could result in cross-contamination [51]. Improvements in infection control procedures in clinical laboratories have been made possible by improvements in BSC technology and design. These developments have improved the containment of infectious agents, reduced the chance of contamination, and improved protection for lab staff [52].

Advancements

Advancements in BSC technology are mentioned in Table 3.

**Table 3:** Advancements in BSC technology BSC: Biosafety cabinet; HEPA: High-efficiency particulate air; ULPA: Ultra-low penetration air; UV: Ultraviolet

Advancements	Description
Improved filtration systems	Modern BSCs are equipped with highly effective, long-lasting HEPA filters. When ULPA filters are used, BSCs can contain particles as small as 0.12 microns even better. A cleaner, safer workplace is ensured by improved filtration technology, which also lowers the risk of infection transmission [53].
Smart sensors and controls	Smart sensors and controls that continuously monitor airflow, filter saturation, and cabinet integrity are present in many more modern BSCs. To guarantee the best performance and safety, these sensors give consumers real-time feedback and warn them of any problems [54].
Energy efficiency	Energy efficiency has been a major priority in BSC design developments, resulting in lower power usage and operational expenses. As a result, BSCs are more cost- and environmentally effective for laboratories [55].
Noise reduction	Modern BSCs are made to be quieter, providing laboratory staff with a less disturbing and more comfortable working atmosphere [56].
Ergonomic design	To promote user comfort over extended working hours, BSCs now have improved ergonomic designs that include movable work surfaces, footrests, and armrests [57].
UV decontamination systems	The inside surfaces of some BSCs are automatically decontaminated between sessions by UV germicidal light. Before beginning new tasks, this function makes sure the workplace is pristine and antiseptic [58].

Contributions to infection control measures

The risk of personnel exposure and cross-contamination is decreased via better airflow patterns, filtration systems, and airtight seals, which help contain infectious agents. The precision and dependability of laboratory results are improved by advances in BSC technology, which guarantees a sterile working environment [59]. Intelligent sensors and ongoing monitoring help to spot possible problems early, enabling quick correction and reducing the risk of contamination. For laboratory workers, ergonomic design and noise reduction help provide a safer and more comfortable work environment that encourages better concentration and adherence to safety procedures [60].

Future developments in BSCs

Additional advances in airflow engineering can result in more effective aerosol and particulate containment, strengthening infection control methods. Innovative filters with even greater efficiency, capable of capturing tiny particles and bacteria, may be produced by non-technological research [61]. Data tracking, remote monitoring, and automatic maintenance planning could all be made possible with the integration of BSCs with laboratory information systems. The interiors may be sterilized more quickly and thoroughly due to improvements in decontamination techniques, such as evaporated hydrogen peroxide [62]. Future BSCs could be made to be more modular and transportable, enabling flexible installation and adjustment to changing laboratory requirements. A wider variety of chemicals and materials may be supported by BSCs, broadening the scope of their applicability in various scientific environments. The integration of robotic and automated technologies within BSCs can help improve operational effectiveness and further reduce the risk of human error [63].

The overall decline in infection rates in clinical laboratory settings is significantly correlated with the application of effective BSC techniques. BSCs produce a controlled and sterile working environment when used correctly and maintained by established guidelines. This reduces the chance of cross-contamination, inhibits the discharge of infectious agents into the lab environment, and reduces the possibility that laboratory staff are exposed to hazardous materials. As a result, there is much less chance of spreading the disease in the laboratory [64]. An obvious decrease in infection incidence has been observed, according to studies and data analysis comparing infection rates before and after the implementation of the BSC guidelines. Following proper hand posture guidelines, using decontamination techniques, performing routine maintenance, and calibrating the BSC, all have a good effect on infection control measures. Laboratories routinely follow the best BSC use procedures, resulting in decreased infection rates and safer working conditions for staff [65].

Future developments in BSCs are outlined in Table 4.

**Table 4:** Future developments in BSCs BSC: Biosafety cabinet

Future developments	Description
Airflow engineering and innovative filters	Additional advances in airflow engineering enhance aerosol and particulate containment, strengthening infection control methods. Non-technological research can produce innovative filters with greater efficiency, capable of capturing tiny particles and bacteria [61].
Data tracking and remote monitoring	Integration of BSCs with laboratory information systems enables data tracking, remote monitoring, and automatic maintenance planning [62].
Improved decontamination techniques	Evaporated hydrogen peroxide and other improved decontamination techniques facilitate quicker and more thorough sterilization of BSC interiors [63].
Modular and transportable BSCs	Future BSCs can be made more modular and transportable, allowing flexible installation and adjustment to changing laboratory requirements [63].
Expanded chemical and material support	BSCs may support various chemicals and materials, broadening their applicability in various scientific environments [63].
Integration of robotic and automated technologies	Robotic and automated technologies within BSCs improve operational effectiveness and reduce the risk of human error [63].

## Conclusions

The BSC plays a crucial role in clinical laboratories as it prevents the spread of infections and protects against emerging diseases. Strict regulations, safety measures, and responsible research practices are essential components of the effective use of these cabinets. Although laboratories are centers of discovery, they also pose risks of accidental release of hazardous agents. Biosafety measures, including biosafety levels, help manage these risks. Biosafety cabinets are indispensable for maintaining safe lab environments, and advanced features and training programs enhance their effectiveness. It is essential to provide adequate training to laboratory personnel to ensure the correct use of biosafety cabinets. In general, biosafety is essential for the protection of individuals, communities, and the environment in laboratory settings.
